# Improving outcomes in Medical Education: A Study of Clinical Cardiovascular Module Performance at Al-Baha Faculty of Medicine

**DOI:** 10.12669/pjms.41.10.12349

**Published:** 2025-10

**Authors:** Yasser Kofiah

**Affiliations:** 1 Yasser Kofiah, Department of Surgery, Faculty of Medicine,Al-Baha University, Al-Baha, Saudi Arabia

**Keywords:** Curriculum Reforms, Learning Strategies, Student Assessment, Progress Test

## Abstract

**Objective:**

To evaluate the factors that contributed to the improvement in Clinical Cardiovascular Module (CCVM) performance at Al-Baha Faculty of Medicine Al Baha University, Saudi Arabia. focusing on Progress Test (PT) results.

**Methodology:**

A retrospective observational study was conducted at Al- Baha Faculty of Medicine, Al Baha University, Saudi Arabia. Two methods were used, combining quantitative data analysis of PT results for CCVM component, with metrics such as average scores and year-wise performance which analyzed across two periods the initial Period 2018 to 2020 where performance was stagnant or declining and the Improvement Period 2021 to 2024 where performance was improving with qualitative data from faculty members and students through faculty interviews and student surveys to explore changes in teaching methods, curriculum reforms and student support systems.

**Results:**

From 2021 to 2024 shows a significant improvement specially in Year three in which the College Average increased from 22.51% in 2018 to 45.25% in 2022 and Year five the Overall Average also rose from 34.13% in 2018 to 47.46% in 2022. This progress in achievement was confirmed by Statistical analyses using Repeated Measures ANOVA that shows p < 0.001, Independent Samples t-test p = 0.002. Qualitative findings highlighted the impact of active learning strategies, integration and enhanced student support systems as the main drivers of improvement.

**Conclusion:**

curriculum reforms and innovative teaching methods as an intervention, improved student performance in CCVM. These findings are valuable for policymakers and medical educators to improve medical students’ outcomes and prepare them for better clinical practice.

## INTRODUCTION

Medical education is the cornerstone of healthcare systems worldwide, as it directly impacts the quality of future healthcare providers and patient outcomes.^1^ In Saudi Arabia medical education has been changing rapidly in the last decade as a result of increasing number of medical colleges numbers.^2^,^3^ and medical education meetings as an educational activity that can play important aspect in providing high quality healthcare teachings,^4^ High-quality medical education directly impacts the safety and effectiveness of patient care as it ensures that graduates are well prepared to meet the complex challenges of healthcare delivery.^5^ The Clinical Cardiovascular module (CCVM) is an important component of the medical curriculum that is because of the global burden of cardiovascular diseases (CVD) such as ischemic heart disease which is the leading causes of death worldwide.^6^,^7^

So, it is essential for medical students to develop a deep understanding of cardiovascular basic medical science and therapeutics for CVD. With rise in risk factors such as hypertension, obesity and sedentary lifestyles,^8^ there is an increasing demand for will-skilled healthcare providers in the management of CVD. So, to achieve this target, medical curricula should integrate committed teaching with practical clinical experiences through interactions with patients during bedside teaching and simulation-based training. In Saudi Arabia Progress test (PT) is a key assessment tool in medical education designed longitudinally across all years of study to assess students’ knowledge and clinical reasoning skills.^9^

In contrast to traditional tests that focus on the content of a specific course the PT assesses cumulative knowledge and competencies expected of a medical graduate providing a comprehensive measure of a student’s development over time.^10^ PT characterized by administered multiple times throughout the medical program, track students’ progress over time cover a broad range of medical knowledge align with the learning objectives and provide individual and group feedback to students helping them identify areas of weakness and strengths for improvement^11^. PT are widely used in Saudi medical colleges to Align with the Saudi Medical Education Directives Framework (Saudi Meds).^12^ It also provides a uniform measure of student performance across institutions promoting quality assurance and accreditation.^10^

At Al-Baha Faculty of Medicine between 2018 and 2020 The CCVM has been one of the most challenging curricula due to stagnation or even declining performance of students. However, since 2021 up to 2024 a remarkable improvement in PT results was noticed. These trends suggest that Targeted interventions were implemented during this period (2021-2024) and lead to improvement in students’ performance.

This study aimed to evaluate the factors that contributed to this notable improvement in Clinical Cardiovascular Module (CCVM) performance at Al-Baha Faculty of Medicine focusing on PT results from 2018 to 2024 concentrating on changes in curriculum design, teaching methodology, and strategy. This was done by analyzing PT results from 2018 to 2024 and insights from faculty staff and students to identify the strategies that have driven this positive change.

The results of this study are expected to contribute to the growing knowledge on effective medical education practices in Saudi Arabian medical colleges and furthermore Al-Baha Faculty of Medicine can serve as a model for other institutions striving to improve their educational outcomes.

## METHODOLOGY

This retrospective observational study was carried out at Al- Baha Faculty of Medicine, Al Baha University, Saudi Arabia. The study examined student performance in the Clinical Cardiovascular module (CCVM) over a seven-year period (2018–2024), comparing progress exam results between the initial period (2018–2020) and the improvement period (2021–2024). In this retrospective study, we used a mixed-methods approach, combining quantitative data (PT results) with qualitative insights PT results and qualitative insights from students and faculty staff. And compare these test results across two periods of time, The Initial Period (2018-2020) characterized by stagnation in performance and the Improvement Period (2021-2024). The qualitative data collected through student surveys and faculty interviews to explore changes in curriculum reforms and teaching strategies.

### Exclusion Criteria:

Students or academic years with missing, students who did not participate in key interventions, students who transferred into or out of the program during the study period, and students who were not enrolled in the CCVM or belonged to non-medical programs.

### Ethical Considerations:

The study has been performed in accordance with the Declaration of Helsinki. The ethical approval was obtained by the Institutional Review Board (IRB) of Al-Baha Faculty of Medicine (IRB/SUR/BU-FM/2024/154, Date: January 12, 2025). All participants (faculty and students) provided informed consent before participating in interviews, surveys, or focus groups.

### Quantitative stage:

PT analysis from 2018 to 2024 to identify trends in student performance in the CCVM component as recorded in Al-Baha Faculty of Medicine academic records.

### Qualitative stage:

Insights from faculty members and students to understand the Factors affecting these trends. Time period divided into two periods, the initial period (2018-2020) which exam performance in the Clinical Cardiovascular module remained relatively low or unchanged, showing minimal year-to-year progress, this is the Period of stagnation in performance and improvement period (2021-2024) marked by a notable increase in student achievement, with higher average scores and improved pass rates. The improvement aligns with the implementation of curriculum reforms and innovative teaching strategies during these years which shows significant improvement in PT results.

### Qualitative Data:

It was collected through semi-structured interviews with faculty members involved in teaching the CCVM. Key topics explored included changes in teaching methods, such as the adoption of active learning and its integration into both medicine and surgery. For instance, Case-Based Learning (CBL) was highlighted as a method where students examine both medical and surgical management of conditions like peripheral vascular disease, comparing treatment options such as pharmacological therapy and surgical interventions. Additionally, Problem-Based Learning (PBL) was used to integrate basic and clinical sciences, with students studying the pathophysiology of conditions like myocardial infarction before applying this knowledge to interpret clinical symptoms and guide treatment decisions, Curriculum reforms e.g., updates in the course content, alignment with PT objectives and Student support initiatives e.g., mentorship programs, academic advising. Surveys and focus group discussions was done with students who completed the CCVM during the study period.

### Statistical Analysis:

Obtained data were presented as average Scores: Mean scores for the CCVM component in each academic year, Overall and College Averages and several statistical methods were used to assess and compare the significance of performance and progress across the two time periods, the initial Period (2018-2020) and the Improvement Period (2021-2024). Statistical analysis was conducted using the SPSS (Version 20) for Windows statistical package. P value <0.05 was considered statistically significant.

## RESULTS

The study included seven years (2018-2024) of PT, observations from analysis show that the performance in the early medical levels years (year one and year two) remained relatively low across all years, with college average ranging from 2.38% to 16.27% and overall average ranging from 5.48% to 14.76%. The college average increased in year 3 from 22.51% in 2018 to 45.25% in 2022, the college average in year four and year five reaching 42.63% in 2021 and overall average rose to 47.46% in 2022, (Table-I).

**Table-I:** PT Performance (2018-2024) Comparison of overall averages and college-specific averages to identify trends in CCVM.

Time period	Year	Year 1 College Average%	Year 1 Overall Average%	Year 2 College Average%	Year 2 Overall Average%	Year 3 College Average%	Year 3 Overall Average%	Year 4 College Average%	Year 4 Overall Average%	Year 5 College Average%	Year 5 Overall Average%
The Initial Period	2018	3.80%	8.74%	10.87%	14.76%	22.51%	23.65%	21.00%	27.54%	28.25%	34.13%
	2019	3.80%	8.74%	10.87%	14.76%	22.51%	23.65%	21.00%	27.54%	28.25%	34.13%
	2020	2.38%	7.92%	8.82%	13.09%	29.38%	24.23%	32.18%	34.88%	32.95%	41.32%
The Improvement Period	2021	4.54%	8.47%	16.27%	17.49%	28.68%	22.20%	42.63%	39.32%	40.86%	46.00%
	2022	6.19%	7.62%	11.92%	17.26%	45.25%	30.12%	33.44%	38.90%	41.66%	47.46%
	2023	8.08%	11.23%	17.16%	19.96%	41.72%	25.62%	27.77%	33.61%	46.69%	43.37%
	2024	5.57%	5.48%	5.81%	8.45%	16.02%	13.64%	25.07%	21.56%	41.27%	33.55%

### The Initial Period (2018-2020):

Characterized by relatively low and stagnant performance. year one and two scores remained low. year three scores showed some improvement in 2020 but remained relatively low. year five overall average in 2020 (41.32%) showed some progress but was still behind later years.

### The Improvement Period (2021-2024):

There is significant progress, there is Higher scores in year three (45.25% and 41.72%) in 2022 and 2023 respectively. overall averages in 2022 was 47.46% in year five, with a notable increase in year four which was 42.63% college average and 39.32% overall average. The overall average in year five is consistent with the upward progress observed in later years. The details of both periods are shown in (Table-I).

Our study found a statistically significant improvement in the mean student results (*p* = 0.001) following the implementation of CCVM changes, with an increase of approximately 10% compared to the initial period, (Table-II). The repeated measures ANOVA results in (Table-III) indicate that there is a statistically significant difference in student performance across the years 2018 to 2024 (*p* < 0.001). This suggests that the implementation of CCVM changes had a measurable impact on student outcomes over time.

**Table-II:** Compare the mean scores of Overall Average across all years (2018-2024) for each academic year.

Test name	Academic Year	Initial Mean (2018-2020)	Improvement Mean (2021-2024)	p-value
chi-square tests	Year 1	6.68%	7.45%	0.120
	Year 2	12.54%	16.89%	0.010
	Year 3	23.45%	32.67%	0.001
	Year 4	27.54%	38.90%	0.002
	Year 5	34.13%	45.32%	0.003
Independent Samples t-test Results	Mean (College Average)	22.34%	35.67%	0.002
	Standard Deviation	8.45	6.78	

**Table-III:** Repeated Measures ANOVA Results.

Source of Variation	Sum of Squares	Degrees of Freedom (df)	Mean Square	F-value	p-value
Within-Subjects (Year)	1200.45	6	200.08	15.67	<0.001
Error	450.30	42	10.72	-	-

## DISCUSSION

Observation after analysis of Student performance in CCVM from 2018 to 2024 reveals that in the early medical levels (year one and year two) performance remained relatively low across all years (2018 till 2024), with college average ranging from 2.38% to 16.27% and overall average ranging from 5.48% to 14.76%. This indicates that students had difficulties in understanding the basic knowledge of the CCVM that remains a challenge for early levels medical students. In year three (level 7 and 8) there is a significant improvement in performance, particularly from 2021 onward. The college average rose from 22.51% in 2018 to 45.25% in 2022, indicating better attainment of the CCVM course principles during the middle level of the curriculum. In the more advanced medical college levels (year four and year five).

The most notable gains were observed, with college average reaching 42.63% in 2021 and overall average rose to 47.46% in 2022. This is showing the impact of target interventions in preparing medical students for clinical practice and advanced CCVM. There is a clear upward trend in performance from 2018 to 2024, with the most significant improvements occurring between 2021 and 2024.This aligns with the implementation of the interventions that was implemented for the CCVM like enhanced student support systems and active learning strategies integration. Faculty and students attributed the improvement to several factors, including the adoption of PBL and CBL, which enhanced engagement and understanding, along with stronger integration between basic and clinical sciences. At, a study evaluating the interdisciplinary integrated Cardiovascular System (CVS) module reported higher student satisfaction compared to the previously implemented temporally coordinated module, although performance was slightly lower, likely due to a more challenging exam.^13^ Similarly, a study at Arabian Gulf University in Bahrain found that integration within the CVS module fostered knowledge, clinical reasoning, and self-directed learning in an enjoyable environment, ultimately supporting satisfactory learning outcomes.^14^

These significant improvements in student outcomes, particularly from 2021 onward directed us to provide a comprehensive understanding of the factors that lead to these improvements and their implications for medical education. The initial period was characterized by stagnant or declining performance, particularly in the early levels (year one and year two). The college average for year one ranged from 2.38% to 3.80%, and the overall average ranged from 7.92% to 8.74%, indicating that students struggled with basic concepts of the CCVM. This aligns with findings from other studies such as Epstein,^15^ which suggest that early-level medical students often face challenges in mastering CCVM^16^ and also aligns with findings of another study from Artino et al,^16^ which suggest that early levels medical students often face challenges in mastering courses like cardiovascular modules due to the high cognitive load and the complexity of its content.^16^

The performance in year three, slightly improved in 2020 college average 29.38%, but the scores remained relatively low compared to the more advanced medical levels (year four and five). These findings are supported by studies by Artino et al^16^ and Wijnen-Meijer et al,^17^ which suggests that mid-level students were not yet achieving the level of mastery needed for clinical practice indicates and that students struggle with integrating the basic concepts of CCVM into clinical reasoning during the middle years of medical school.^16^,^17^

Similarly, the performance in year five showed gradual improvement, with the overall average increasing from 34.13% in 2018 to 41.32% in 2020, but this was still below the levels achieved in the improvement period which shows a better achievement due to new teaching strategies and better preparation for clinical practice. Studies like Van der Vleuten et al.^18^ have shown that final level medical students often require more targeted preparation for clinical practice including exposure to real clinical scenarios and good assessments.^19^

In the improvement period, we noticed a significant upward progress in performance, particularly in year three and year five, as shown in PT results analyses, college average for Year three increased from 22.51% in 2018 to 45.25% in 2022, indicating better mastering of CCVM. This improvement is likely, as shown by studies by Schuwirth^19^ and van der Vleuten,^18^ to be due to the implementation of new learning strategies and enhanced student support systems, which have been shown to improve student performance.^18^ In Year five, the overall average rose to 47.46% in 2022, due to better preparation for clinical practice. Finally, the end results of this study are supported by many previous studies in the field of medical education and progress tests. such as a study by (Wijnia L et al.^20^ ) that adopt PBL shown to enhance student understanding.^20^

The significant improvement in year three performance during the improvement period supports this theory, as active learning strategies were a key intervention. Another study by Elendu C, et al, that linked the use of virtual simulations and online resources to improved performance in medical education.^5^ This likely contributed to the improvement, particularly in year four and year five, where students benefited from enhanced bedside teaching. In the field of medical education this study highlights the importance of aligning the curriculum with SaudiMEDs as a national competency standard with integrating active learning strategies. Also, the role of mentorship programs and academic advising in improving student performance. Finally, the use of virtual simulations and e-learning platforms in CCVM should be expanded to other modules, as they have proven effective in enhancing student performance.

### Strengths:

Analysis of the seven-year period provides a comprehensive view of performance progress and the impact of interventions over time. By analysis of PT data and qualitative data we can understand the factors driving this improvement. The findings provide targeted recommendations for sustaining and enhancing medical education outcomes.

### Limitations:

The study is limited to Al-Baha Faculty of Medicine, which may affect the generalizability of the results. Also, the Qualitative data from faculty members and students may be influenced by social desirability bias and the difference in student demographics or external events as the COVID-19 pandemic which may have influenced the results.

## CONCLUSION

Analysis of progress exam results from 2018 to 2024 demonstrated a clear improvement in student performance in the Clinical Cardiovascular module at Al-Baha Faculty of Medicine, particularly from 2021 onward. This improvement coincided with curriculum reforms and the implementation of innovative and creative teaching methods, which were associated with higher average scores and better overall outcomes.
